# Gradual introduction of carbon allowance auctions facilitates sustainable emission reductions in the power sector

**DOI:** 10.1016/j.isci.2026.116241

**Published:** 2026-06-08

**Authors:** Zhao-Yuan Li, Lu-Tao Zhao, Zhi Qu, Zhe-Yi Chen, Rui-Xiang Qiu, Xing-Yu An, Dai-Song Wang

**Affiliations:** 1 Center for Energy and Environmental Policy Research, Beijing Institute of Technology, Beijing 100081, China; 2 School of Management, Beijing Institute of Technology, Beijing 100081, China; 3 Tangshan Research Institute, Beijing Institute of Technology, Tangshan 063000, China; 4 Energy Economics Institute, China National Offshore Oil Corporation, Beijing 100013, China

**Keywords:** Environmental science, Energy engineering, Economics

## Abstract

Achieving deep decarbonization of the power sector is essential for China’s carbon neutrality goal and global climate mitigation. However, the coordination among emission reduction effectiveness, carbon market stability, and energy security remains unclear. This study develops a bottom-up multi-agent simulation model, Electricity and Carbon Coupling Multi-Agent System (ECMAS), integrating the power market with primary and secondary carbon markets to capture the adaptive behaviors of 2,241 heterogeneous power enterprises under alternative carbon market designs. Four policy scenarios are simulated to evaluate different pathways of quota tightening and auction introduction. Results show that rapidly synchronizing quota reductions with high auction shares imposes excessive carbon pressure, leading to carbon price collapse, premature fossil capacity retirement, and supply risks. In contrast, gradually introducing auctions alongside smooth quota tightening stabilizes carbon prices, supports phased low-carbon investment, and achieves sustained emission reductions. These findings provide evidence-based guidance for improving China’s carbon market and offer transferable insights for global carbon market design under deep decarbonization.

## Highlights

• Developed the Electricity and Carbon Coupling Multi-Agent System (ECMAS)
• Introduced dynamic quota reduction ratios and auction proportion scenarios
• Simulated the decision-making processes of power industry enterprises in China
• Analyzed the trade-offs between the costs and effectiveness of emission reductions

## Graphical abstract

**Figure:**
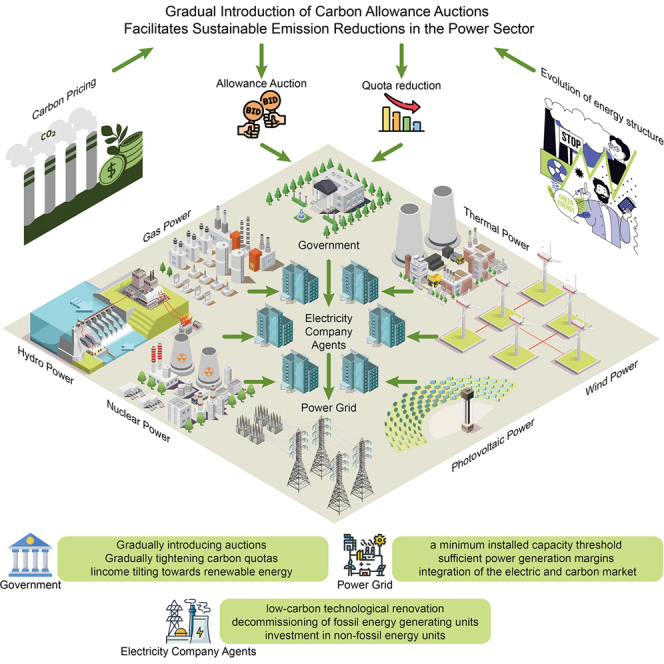

## Introduction

### Climate goals and carbon neutral system engineering

Climate change is a significant challenge that the world faces together. The latest research from the Intergovernmental Panel on Climate Change (IPCC) Sixth Assessment Report indicates that to achieve the goal of limiting global warming to no more than 1.5°C, global CO_2_ emissions need to be significantly reduced in the next 10 years.^1^ The outcomes of COP30 reaffirm that robust carbon markets have become a central mechanism for driving deep and verifiable global emission reductions. China’s national emissions trading system (ETS)—anchored by the power sector, the world’s largest single source of CO_2_ emissions—emerges as a critical pillar in global decarbonization.

The reduction of emissions in the power industry is a key area for achieving carbon neutrality goals. The power industry is characterized by a large emission base, significant reduction potential, and multiple pathways for emission reduction. Firstly, as a traditional carbon-intensive industry, the power sector is the largest single carbon-emitting industry in China, accounting for 41% of the country’s total carbon emissions as of 2020.^2^ Secondly, the carbon emissions from the power industry are concentrated and easy to control, making it the most feasible sector for replacing fossil energy with non-fossil energy. Thirdly, there are diverse pathways for the low-carbon transition of the power industry, mainly including the development of renewable energy generation technologies, improving the efficiency of fossil energy, and developing carbon capture and storage (CCS) technologies.^3^ Moreover, the clean transformation of the power industry is also closely related to energy conservation and emission reduction in other carbon-intensive industries such as steel, chemicals, and building materials,^4^ contributing to emission reductions from multiple aspects. Therefore, promoting the low-carbon transition of the power industry is a key pathway to achieving the dual carbon goals.

### The development of China’s electricity power market and carbon market

The ongoing reform of the electricity market has increasingly positioned the market as a pivotal mechanism for the allocation of electricity resources, thereby enhancing the efficiency of electricity production and distribution. The national market-based trading volume of electricity surged from 1.1 trillion kilowatt-hours in 2016 to 6.2 trillion kilowatt-hours in 2024, with the share of total electricity consumption escalating from 17% to 63%. Concurrently, the number of registered trading participants expanded dramatically from 42,000 in 2016 to 816,000 in 2024, encompassing the entire spectrum of generation, sales, distribution, and consumption. An initial market-oriented pricing mechanism that accommodates both price increases and decreases has been established, leading to a gradual liberalization of prices in competitive sectors.

The new power system, characterized by the synergistic interplay of policies, technological advancements, and market mechanisms, significantly enhances the efficient utilization of renewable energy resources. In 2024, the National Energy Administration released the “2024 Energy Work Guidance Opinion,” which explicitly aims to elevate the share of non-fossil energy power generation capacity to approximately 55%. Furthermore, it sets a target for wind and solar energy to constitute over 17% of the nation’s total power generation. The establishment of this new power system, initially proposed in 2021, has fostered the comprehensive integration of renewable energy into market transactions.

### The carbon market is regarded as an efficient mechanism for achieving climate goals

In recent years, governments worldwide have increasingly implemented three primary types of environmental regulations to mitigate carbon emissions, as summarized in Table 1.^10^^,^^11^ Among these, market-based regulatory instruments have gained widespread adoption due to their effectiveness and flexibility.^12^^,^^13^^,^^14^ In particular, the carbon finance system—exemplified by carbon emission trading schemes—has emerged as a key market mechanism. Theoretically, following the allocation of emission allowances in the primary carbon market, enterprises with net emissions can achieve cost-effective compliance by purchasing allowances in the secondary market at a price lower than their internal abatement costs. Conversely, entities with surplus allowances can realize financial gains by selling these allowances at a price exceeding their abatement costs. The centralized market transactions enable the reallocation of carbon emission rights to sectors where they are most efficiently utilized, thereby promoting overall emission reductions.^15^

**Table 1:** Classification of environmental regulations and examples

Regulation Types	Examples	Reference
Command-and-control regulation	environmental protection law, mandatory emission standards, investment in environmental pollution control, pollutant discharge permit system, environmental administrative penalties, etc.	Zhao et al.^5^
Market-based regulation	environmental taxes and fees, emission trading, pollutant discharge fees, ecological compensation, etc.	Du et al.^6^; Du et al.^7^
Voluntary regulation	information disclosure, environmental agreements, media pressure, etc.	Arimura et al.^8^; Kathuria et al.^9^

To promote emission reductions in the power sector, China has implemented a series of targeted energy policies. These include administrative measures to phase out outdated production capacity, the establishment of energy efficiency standards to facilitate the upgrading of coal-fired power plants, investment subsidies for energy-saving initiatives, and price incentives for renewable energy projects. However, the extensive use of fiscal subsidy policies imposes increasing financial pressure on both central and local governments, raising concerns about long-term sustainability. Meanwhile, the marginal cost of administrative interventions continues to rise, while their marginal effectiveness steadily declines.^16^ In this context, the development of a national carbon market will advance the low-carbon transformation of China’s power industry.

The establishment of the national carbon market reflects China’s transition in climate change policy mechanisms—from a primary reliance on administrative measures and financial subsidies toward the adoption of carbon pricing.^16^ Drawing on international experiences in carbon market development, a gradual reduction in emission allowances and the introduction of paid allocation are recognized as key design elements in effective carbon market frameworks.^17^ The allowance reduction mechanism enhances emission reduction ambition and reinforces allowance scarcity by progressively lowering the total allowable emissions annually. Meanwhile, the paid allocation mechanism, particularly through auctioning, not only increases the opportunity cost of emissions but also offers significant advantages such as price discovery and improved resource allocation efficiency.^18^^,^^19^ According to the “Measures for the Management of National Carbon Emission Rights Trading (Trial),” China will adopt a hybrid allowance allocation approach, primarily based on free allocation, with the phased introduction of paid allocation at an appropriate stage.

Therefore, designing a mechanism that simultaneously considers both emission reduction effects and economic benefits, while implementing a dynamic reduction of quotas and an increase in the auction ratio, has become a key issue in the development of the carbon market. At the same time, we also need to take into account various factors such as whether this policy is beneficial to the development of new energy, national energy security, and the reliability of the power system.^20^

### ABM enables the identification of emergence properties through a bottom-up approach

The shift toward an electric-carbon coupling has introduced a multitude of technical, social, economic, political, and environmental challenges,^21^ particularly in accurately comprehending and effectively characterizing the intricate interactive coupling and complexity between socio-economic systems and natural environmental systems.^22^ In the past, a large number of studies on the low-carbon transition of the electricity sector focused on the use of optimization models^23^^,^^24^^,^^25^ and equilibrium models.^26^^,^^27^^,^^28^ There are also studies that analyze the impact of the carbon market on the electricity market and the coupling effect between the two markets.^29^^,^^30^ However, under the disequilibrium condition, how to react to the market through the behavioral strategy of each agent should be simulated by the agent-based model (ABM).^31^^,^^32^

After the implementation of the cap-and-trade mechanism, it is essential to illustrate the complex decisions made by entities in the electricity and carbon markets. To account for interaction effects, heterogeneity, bounded rationality, and learning evolution among these entities, the ABM was widely adopted due to its effectiveness in capturing the emergent properties of complex systems from a bottom-up perspective.^33^

Regarding the research on emission reduction policies in the power industry, the existing studies mainly focus on the evolution of the power structure, support policies for new energy, demand response, risks and technological preferences, as well as efficiency gaps in imperfect markets. Chen et al.^34^ set the generator, wholesaler, and government agent, simulating the impacts of carbon tax and on-grid power price (FIT) policies on China’s power generation structure and carbon emissions during 2010–2050. The FIT policy will promote investment in wind and solar power but will crowd out investment in gas and nuclear power, rather than replace coal. Carbon tax policies, however, are more effective in reducing emissions and incentivizing a variety of low-carbon power generation technologies.^34^ Wu et al.^35^ developed an ABM for China’s Renewable Portfolio Standard, capturing the complex interactions and decision-making behaviors of obligated entities at the micro level as they coordinate among three compliance pathways: electricity consumption, tradable green certificate (TGC) consumption, and consumption above allowance (CAQ) transactions. From the perspective of multi-market coupling, the model illustrates the dynamic, emergent interactions across the power market, TGC market, and CAQ market at the macro level.^35^ Sridhar et al. incorporated relevant economic, social, and behavioral parameters influencing consumer participation demands and examined the significant role of residential consumers’ demand response—through the utilization of household loads—in advancing the sustainable transformation of energy systems.^36^

The aforementioned research focused on the impact of various policies and did not investigate the heterogeneous preferences of power generators. Chen et al.^37^ examined the impact of power generation companies’ risk and technology preferences on the long-term low-carbon transition through a combination of multi-agent modeling and Monte Carlo simulation. It was found that risk aversion and technology preference significantly promote the low-carbon transformation of the power industry, and there is a synergistic effect between the two.^37^ Torralba-Díaz et al. integrated an optimization model—based on the theoretical assumptions of perfect competition and perfect foresight in the electricity market—with an agent-based simulation model that accounts for market inefficiencies, to examine the economic disparities between a theoretically cost-optimal power system and a power system operating under imperfect market conditions.^38^

ABM provides a new solution for studying the complex interaction behaviors of power enterprises under the constraints of the carbon market. The scholars conducted their research by examining carbon price mechanisms, analyzing carbon allowance allocation policies, and investigating enterprise decision-making behaviors under carbon regulation. Richstein et al. examined the policy options under five different lower and upper limits of carbon prices, with random input parameters for different electricity demands and fuel prices. The results indicated that the price floors mitigated the fluctuations in carbon prices and the occurrence of carbon shortages and lowered the average cost for consumers, leading to a more sustainable decarbonization path.^39^ The price ceiling could protect consumers from extreme price shocks. Möst et al.^40^ found that the design of allowance allocation is critical to the effect of emission reduction in the power sector. Free allocation leads to increased investment in high-carbon technologies and higher long-term emission reduction costs. The auction mechanism is more effective in promoting the low-carbon transition, but the short-term electricity price has increased significantly.^40^ Wei et al. conducted a simulation of transactional interactions among diversified enterprises and found that, due to the heterogeneous characteristics and marginal abatement cost (MAC) curves across industries—specifically, the significant variation in MACs required to achieve the same percentage of emission reduction—regulated firms adopt different compliance strategies under the ETS, reflecting strategic trade-offs and decision logic that shape the emergent dynamics of the ETS market.^41^

### Carbon allowance auction

For different implementation paths of carbon allowance mechanisms, most governments currently set a fixed rate of quota reduction. However, Jeitschko et al.’s equilibrium analysis indicates that setting a flexible upper limit for the carbon allowance supply in each period can be a better alternative to reduce price fluctuations.^42^ Xu and Xu constructed an energy trading platform supply chain consisting of three power plants, one energy trading platform, and one power retailer. Their analysis shows that by reducing the free allocation coefficient of carbon allowances and simultaneously increasing the annual quota reduction ratio, carbon costs will be more fully passed on to electricity prices.^43^ Liu et al. found through a multi-objective linear programming study that when the auction ratio increases, the carbon emission reduction cost also increases, and the growth rate increases accordingly.^44^ Further, Zhang et al. combined the multi-objective particle swarm optimization (MOPSO) algorithm with the TOPSIS method and suggested that the optimal initial auction ratio of carbon quotas in the power generation industry is within the range of 4%–5.5%.^45^

For the impact of auctions, scholars generally believe that in a situation of compensated allocation, along with heightened consumer low-carbon awareness, government subsidies, and a reduction in the carbon emission cap, the enterprises under regulation tend to reduce carbon emissions.^46^ An increase in the auction ratio has a relatively small impact on the optimized energy production structure, especially on the share of renewable energy resources.^47^ Auctions can lead to the risk of carbon leakage, but exemption from auctions is unlikely to reduce carbon leakage; instead, it may exacerbate competitive distortions.^48^

For different auction methods, Yang et al. determined the optimal carbon quota price through the Nash bargaining solution (NBS), proving that compensated allocation can reduce carbon emissions in the integrated energy system to a certain extent.^49^ Chen and Ma set up a system technology adoption model with heterogeneous agents, in which the transaction price and transaction volume are achieved through repeated Walrasian auctions at different times.^50^ However, in the real market, investors may tend to provide false supply and demand disclosures. Yang et al. proposed a new carbon quota trading model based on a two-stage sealed-bid auction, proving that it plays a crucial role in promoting the internal carbon trading system and reducing the carbon emission potential of the integrated energy system.^51^ Luo et al. established a fixed-price trading mode considering actual carbon emissions; for Chinese Certified Emission Reduction (CCER) from hydrogen and other renewable energy sources, a trading mechanism based on a Vickrey auction was introduced, and it was proved to have advantages in economic performance and carbon emission reduction.^52^

When considering the income redistribution through carbon quotas, Gavard et al. found that using the auction revenue from the EU Emissions Trading System (EU ETS) to reduce national taxation would lead to a 1.8% increase in ETS carbon prices but a 5.9% decrease in non-ETS carbon constraints.^53^ Wang and Duan studied consignment auctions and found that compared to free distribution, consignment auctions can achieve higher and more stable carbon prices without significantly increasing the costs borne by enterprises, increase the liquidity of the ETS, and stimulate technological progress in enterprises.^54^ Compared to auctions, consignment auctions reduce the fluctuations in the commodity market induced by the ETS, which is conducive to the smooth operation of the ETS. Further, Guo et al. constructed a simulation framework for an incentive-oriented integrated market of power-CET-TGC based on multi-agent deep reinforcement learning and found that under the short-term goal of “carbon peak” that requires economic benefits and stable emission reduction, it is recommended to adopt the consignment auction mechanism, while under the long-term goal of “carbon neutrality” that requires large-scale emission reduction, it is recommended to adopt the traditional auction mechanism.^55^

In conclusion, scholars’ research still lacks a roadmap study on the innovation of China’s carbon market mechanism. We will conduct simulation analysis on the dynamic setting of the quota reduction ratio and the auction proportion.

### Marginal contribution

Recent developments at COP30 reaffirm the carbon market as a central instrument in global climate governance, with countries shifting from rule-setting to a “deep implementation phase formula 4” focused on actual emission outcomes. New initiatives—such as Brazil’s proposal for a Global Carbon Market Integration Framework and the launch of the Open Coalition on Compliance Carbon Markets—highlight accelerating efforts to enhance linkage, transparency, and cross-border coordination in carbon pricing. Against this backdrop, China’s national ETS plays a pivotal role, not only as the world’s largest emerging carbon market but also as the primary mechanism to drive low-carbon transformation in the power sector, which remains the country’s largest and most controllable source of emissions. Yet China is still navigating the transition from predominantly free allocation toward more market-based mechanisms, and the optimal design of quota tightening and auction introduction has not been systematically evaluated. This gap underscores the necessity of our study and highlights the importance of developing evidence-based pathways for China’s carbon-market reform and power-sector decarbonization.

In summary, we employed an ABM to address emission reduction challenges in the power sector within the context of the carbon market, making the following contributions: first, using actual data from China’s power industry, we developed a multi-agent electricity-carbon coupling model that accounts for the complex interactions among power companies across three market transactions—the power market, primary carbon market, and secondary carbon market—during their participation. The entire decision-making process was divided into six modules over a 1-year time frame, accurately simulating the real decision-making pathways of enterprises. Second, drawing on the experience of the EU ETS, we introduced dynamic quota reduction ratios and progressively increased auction proportions to simulate the impact of advancing carbon market mechanisms in China under various scenarios, identifying the optimal improvement strategy. Third, we examined the trade-offs between power market and carbon market performance, the costs and effectiveness of emission reductions by enterprises, as well as the evolving structure of China’s power generation capacity, and offered relevant policy recommendations for stakeholders including the government, power grid operators, and power companies.

The remainder of the paper is organized as follows: The results section presents the simulation outcomes. The discussion section further interprets the findings, summarizes the study, and outlines the key policy implications. The STAR Methods section presents the Electricity and Carbon Coupling Multi-Agent System (ECMAS), as well as the calibration and configuration for simulation.

## Results

### Scenario setting

To more accurately project the future trajectory of China’s national carbon market, we take into account the benefits of the EU carbon market, which include its early establishment, extensive data, and large scale. The construction experience of the EU carbon market offers valuable insights for China.^56^ We have compiled essential parameters from the EU ETS (ETS), such as the annual quota reduction ratio, auction ratio, sectors involved, and thresholds for its four phases, drawing from the EU’s Fit for 55 package and ICAP’s Emissions Trading Worldwide Status Report 2025. These parameters will inform the quota reduction and auction advancement strategy for this model, aiming for a 100% auction ratio by 2034, in alignment with the ultimate goals of Fit for 55.

**Figure 1:**
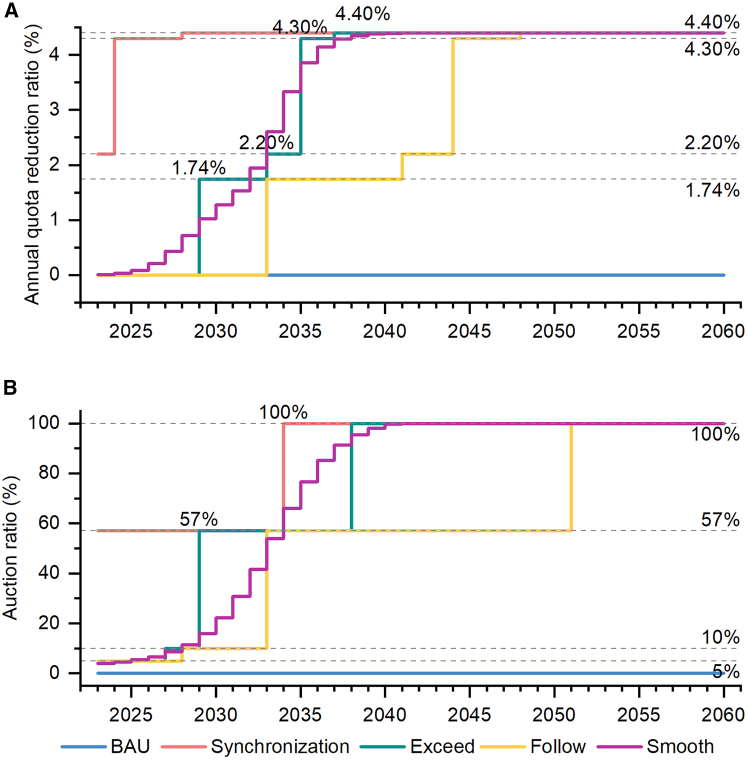
Dynamic setting of annual quota reduction ratio and auction ratio (A) Dynamic setting of annual quota reduction ratio under four scenarios. (B) Dynamic setting of auction ratio under four scenarios.

According to Table 2, we set four scenarios: synchronization, exceed, follow, and smooth. The dynamic setting of the annual quota reduction ratio and auction ratio is shown in Figure 1. The EU ETS is used in this study as a comparative reference for scenario construction rather than a policy template for China. This is necessary given the substantial differences between the two systems in institutional settings, market maturity, and electricity market structures. Institutionally, the EU ETS operates under a highly market-oriented and rule-based framework, whereas China’s ETS is still in a transitional stage with stronger administrative guidance and a dominant reliance on free allowance allocation. In terms of market maturity, the EU ETS has developed over multiple phases with stable price formation, high liquidity, and broad participation, while China’s market remains relatively early stage, with limited coverage, lower trading activity, and evolving price discovery. Regarding electricity market structures, the EU power sector is largely liberalized, enabling effective carbon cost pass-through, whereas China’s system is still under reform, with regulated pricing, significant state ownership, and incomplete spot and ancillary service markets, which may weaken or delay this transmission. Therefore, the EU ETS serves as a conceptual benchmark to explore policy coordination mechanisms.

**Table 2:** The characteristics of each phase of EU ETS

	Phase 1	Phase 2	Phase 3	Phase 4	Goal
Time span	2005–2007	2008–2012	2013–2020	2021–2030	2034
Annual quota reduction ratio	0	0	1.74%	2.2% (2021–2023) 4.3% (2024–2027) 4.4% (2028–)	–
Auction ratio	5%	10%	57%	57%	100%
Sectors and thresholds	power stations and other combustion installations with >20 MW thermal rated input, some industries (various thresholds)	aviation: international aviation (2012)	more industry sectors: carbon capture and storage installations, production of petrochemicals, ammonia, nonferrous and ferrous metals, gypsum, aluminum, as well as nitric, adipic, and glyoxylic acid (various thresholds)	maritime: all large ships (of 5,000 gross tonnage and above) entering EU ports (2024)	–

Synchronization: follow up on the EU scenario at any time.

Exceed: advance auctions based on half the time span of each EU phase.

Follow: advance auctions based on the same time span of each EU phase.

Smooth: integrate the first three scenarios and advance using a smooth curve.

### Carbon market performance

**Figure 2:**
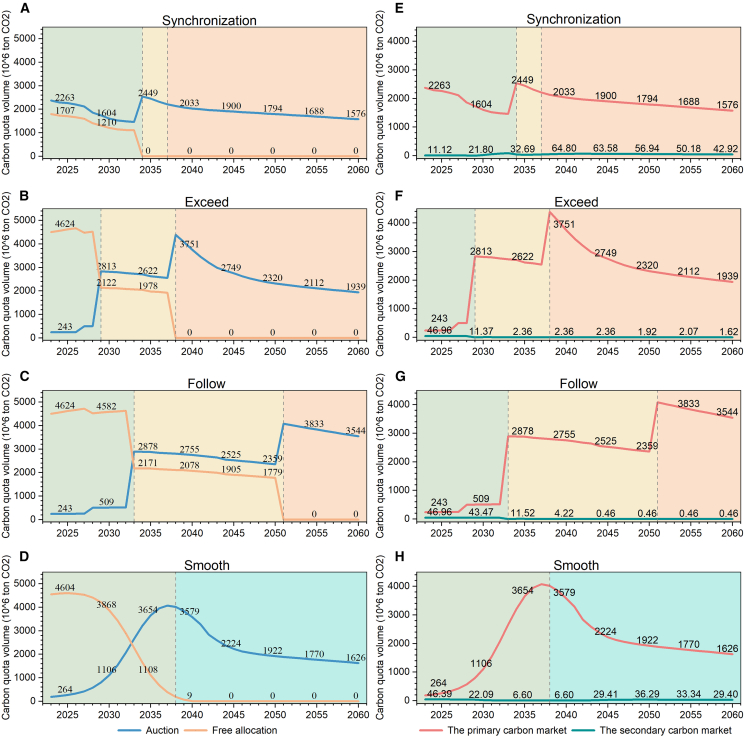
The allocation volume and trading volume of carbon allowances under various scenarios (A–D) The allocation volume of carbon allowances. (E–H) The trading volume of carbon allowances. The dotted lines indicate the years when each policy parameter was increased.

After setting the dynamic curves for each scenario, the carbon allowance volume of the national carbon market is shown in Figure 2. It can be seen that the change in the allowance volume is consistent with the setting of the scenario curves, and it decreases year by year according to the dynamic allowance reduction ratio, indicating the validity of the model setting. It is particularly noteworthy that in the synchronization scenario, when the auction ratio initially increased from 57% to 100% in 2034, there was a significant fluctuation in the trading volume of the carbon market. Furthermore, because the allocation was based on the benchmark method, this ultimately caused fluctuations in the volume of carbon allowances. The specific reasons for the transmission will be discussed in the following analysis.

Regarding trading volume, as the quota reduction ratio increases, demand in the secondary carbon market rises, causing the demand-to-supply ratio to grow significantly and resulting in greater market volatility. Secondly, following the introduction of auctions, the transaction volume in the secondary carbon market increases compared to the free allocation scenario. Additionally, the demand-to-supply ratio in the secondary market decreases relatively, and the degree of fluctuation is also mitigated. Under the synchronization scenario, the cumulative carbon trading volume reaches 1,998.53 million tons, surpassing the cumulative trading volumes of the exceed, follow, and smooth scenarios, which are 355.01 million tons, 562.41 million tons, and 632.82 million tons, respectively. This indicates that the introduction of the auction mechanism has injected new vitality into the carbon market, which is conducive to stabilizing the secondary market, playing a role in price discovery, and promoting the healthy development of carbon market transactions.

Regarding auction volume, auctions dominate trading in the carbon market. Notably, in the concurrent scenario, the overly aggressive introduction of auctions led power enterprises to temporarily shift toward low-carbon technology upgrades and reduce power generation to lower emissions. In the long term, they further decreased emissions by cutting investments in fossil fuel units. As a result, the industry’s overall emission benchmark declined, and the annual quotas were significantly reduced. Under the synchronization scenario, the cumulative carbon auction volume reaches 68.41 billion tons, lower than the cumulative auction volumes of the exceed, follow, and smooth scenarios, which are 101.80 billion tons, 89.70 billion tons, and 100.47 billion tons, respectively. It can be inferred that introducing auctions too aggressively may result in reduced industry output and excessive market reactions, thereby causing issues related to energy security and economic development.

### Carbon prices and unit costs

**Figure 3:**
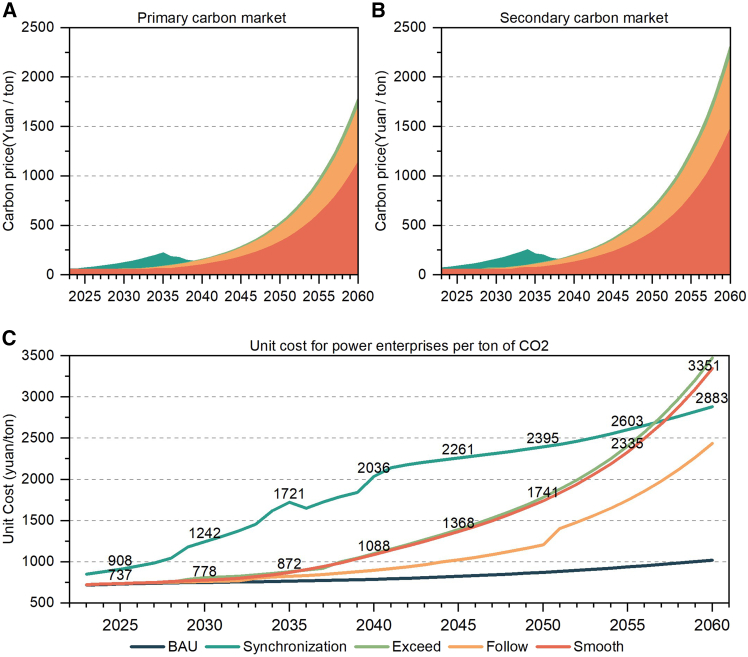
Carbon prices under various scenarios (A) Carbon price in primary market. (B) Carbon price in secondary market. (C) Unit cost for power enterprises per ton of _CO₂_.

Regarding carbon prices, a higher proportion of auctions and a faster rate of quota reduction lead to a more rapid increase in carbon prices, which tend to follow an “L-shaped” curve, as shown in Figures 3A and 3B. In the early stages, the rate of increase is slower and the price rise is modest; in the later stages, the increase accelerates significantly and becomes much more pronounced.

Regarding the carbon price, the synchronization scenario exhibited the highest rate of carbon price increase during the initial stage. However, by 2035, the carbon market experienced severe fluctuations due to an excessively high quota reduction ratio and auction ratio, which had imposed significant pressure on the power industry earlier. Additionally, the sudden increase in the auction ratio to 100% in 2035 exacerbated the situation. Power enterprises were unable to withstand the emission reduction pressures and consequently reduced production. As a result, the demand for carbon quotas declined, causing the carbon price to drop and ultimately leading to the collapse of the carbon market.

Under the smooth scenario, the carbon price in the primary market will be 142 yuan per ton in 2040 and 1,678 yuan per ton in 2060. In contrast, under the exceed scenario, it will be as high as 151 yuan per ton in 2040 and 1,778 yuan per ton in 2060. It can be seen that in the smooth scenario, under the same advancement mechanism innovation as the exceed scenario, a lower carbon price is observed. Analyzing the determinants of the price variable in the model reveals that the smoothly implemented policy mechanism enables power companies to gradually advance low-carbon technology upgrades, proactively adapt to the pressure of reduced carbon quotas, and make investments earlier in non-fossil energy capacity. Ultimately, this allows them to maintain rational decision-making in the carbon market and avoid excessive demand disrupting the market. Correspondingly, the carbon prices in the secondary market under the smooth scenario will be 185 yuan per ton in 2040 and 2,182 yuan per ton in 2060. These prices are slightly higher than those in the primary carbon market, reflecting enterprises’ compliance requirements and transactional frictions.

According to the set conditions of the main action, at the beginning of each period, each power enterprise measures the amount of carbon quotas to purchase based on its own low-carbon transformation status. In this context, within a fully market-based system, the carbon price tends to equal the unit cost of emission reduction, with no significant difference between the two. In this paper, we calculate the unit cost of the power enterprise by dividing the total cost of the enterprise by its carbon emissions. As shown in Figure 3C, under the synchronized scenario, the unit cost for power companies increases rapidly and remains high until 2057, placing significant pressure on these companies. In contrast, the other three scenarios show a slower rate of increase and eventually surpass the synchronized scenario after 2057. This is due to the higher total emission reductions at the end and the resulting increase in marginal abatement costs.

### Evolution of energy structure in power industry

**Figure 4:**
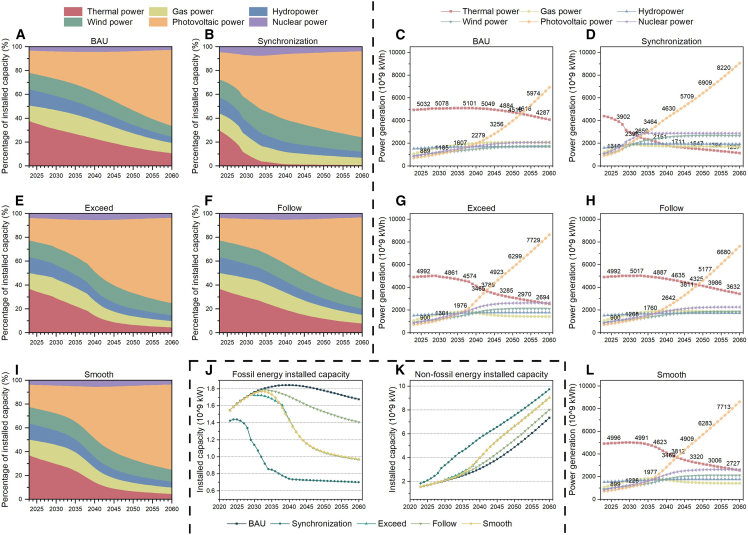
Installed capacity and power generation (A, B, E, F, and I) Percentage of installed capacity. (C, D, G, H, and L) Power generation. (J and K) Comparison of installed capacity between fossil and non-fossil energy.

The overall trend of a decline in the proportion of thermal power installed capacity and an increase in the proportion of non-fossil energy installed capacity is consistent across all scenarios. The earlier the auction is introduced, the faster the installed capacity of non-fossil energy increases, as shown in Figure 4.

Under the synchronization scenario, due to continuous carbon pressure, particularly the sharp increase in 2035, the share of thermal power plants has almost reached zero by 2040, and the power generation from thermal power plants has rapidly declined. This presents a significant challenge to the power system and national energy security and does not align with the current realities. This result arises from a stylized setting without explicit representation of system flexibility, storage deployment, or capacity adequacy constraints. Instead, it highlights the potential risks associated with overly aggressive policy coordination. It suggests that the rapid implementation of carbon market auctions may not be suitable given the national context. In comparison, the smooth scenario achieves the best non-fossil energy substitution, second only to the synchronization scenario, but it also possesses many advantages such as trading volume and carbon pricing, as previously described. Ultimately, by 2060, in the smooth scenario, the proportion of non-fossil energy installed capacity will decrease by 8.86% compared to the business-as-usual (BAU) scenario, and the proportion of electricity generation will decrease by 12.20%. At that time, the proportion of new energy installed capacity will reach 90.29%, and the proportion of electricity generation will reach 79.18%.

### Electricity industry carbon emission reduction

**Figure 5:**
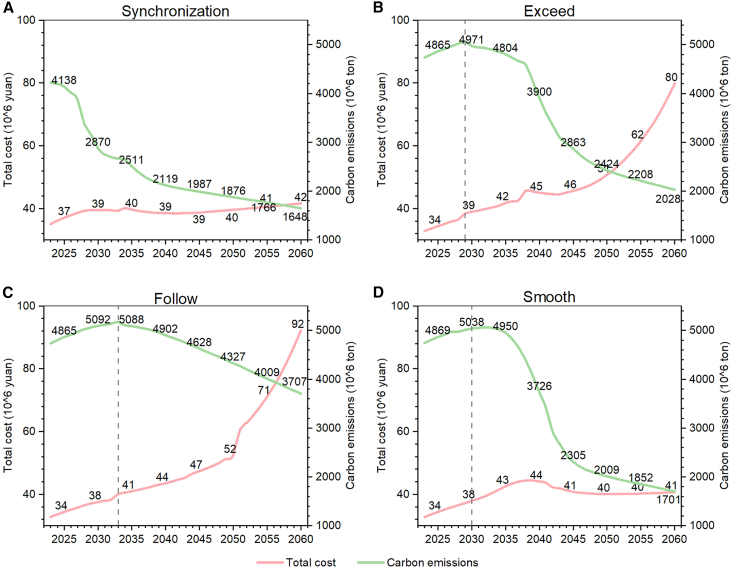
Carbon emissions and total costs in the power industry under various scenarios (A) Carbon emissions and total costs in the power industry under the synchronization scenario. (B) Carbon emissions and total costs in the power industry under the exceed scenario. (C) Carbon emissions and total costs in the power industry under the follow scenario. (D) Carbon emissions and total costs in the power industry under the smooth scenario.

As shown in Figure 5, all four scenarios can achieve the carbon peak target by around 2030. The exceeding scenario will peak in 2029, while the smooth scenario will peak in 2030, aligning with the dual-carbon goals proposed by the government. The follow scenario will peak in 2033. Thus, a comprehensive comparison of the emission reduction outcomes and unit emission reduction costs across various scenarios reveals that, in terms of emission reduction effectiveness, introducing auctions early and advancing quota reductions in synchronization scenario enables the power industry to reach the carbon peak sooner and achieve greater cumulative carbon reductions. Cumulative carbon emission reductions will reach 102.55 billion tons in the synchronization scenario and 37.77 billion tons in the smooth scenario, with the smooth scenario showing the second-best emission reduction performance among all scenarios.

Regarding cumulative emission reduction costs, under the smooth scenario, the cumulative emission reduction cost by 2060 is 924 billion yuan, which is only 2.28% higher than that of the follow scenario, while the cumulative emission reduction increases from 15.22 billion tons to 37.77 billion tons, which is an increase of up to 148.19%. Consequently, when both emission reduction effectiveness and associated costs are taken into account, the smooth scenario emerges as the best option.

In conclusion, the smooth scenario has prevented abnormal market fluctuations caused by excessive carbon pressure in the carbon market, stabilized a relatively reasonable carbon price, maintained the normal production of power enterprises, and effectively promoted the transformation of low-carbon technologies and the investment in non-fossil energy units, as well as the retirement of fossil energy units. It has ensured the stable operation of the power system and national energy security, achieving a balance between emission reduction volume and cost. Therefore, we believe that in the process of increasing the auction ratio and quota reduction ratio, the gradual introduction of carbon allowance auctions facilitates the sustainable emission reduction in the power sector.

## Discussion

We have developed the multi-agent model ECMAS to assist power enterprises in reducing emissions. The model represents three markets: the power market, the primary carbon market, and the secondary carbon market. It includes three types of entities: the government, the power grid, and 2,241 power enterprises. The model spans the period from 2023 to 2060. In each period, six modules—electricity production, electricity trading, low-carbon technology transformation, carbon allowance allocation, carbon allowance trading, and unit investment and retirement—are included, and the dynamic interactions from the bottom up among enterprises reveal the emergent characteristics of the power industry’s participation in carbon market transactions. Based on these insights, we have developed four scenarios and identified the optimal carbon market mechanism design to facilitate inter-plant collaboration within the power industry. The following conclusions have been drawn.

Overly aggressive carbon constraints and auction promotion can undermine market stability and energy security. In the synchronization scenario, the auction ratio and quota reduction ratio were simultaneously and rapidly increased, leading to severe short-term fluctuations in the carbon market. After the auction ratio abruptly rose to 100% in 2035, power enterprises faced excessive emission reduction pressure, forcing them to reduce output, decrease investment, and accelerate the retirement of fossil fuel units. This caused a sharp decline in quota demand and a collapse in carbon prices. Although this scenario achieved the highest cumulative emission reduction (1,02.55 billion tons), it was accompanied by volatile transaction volumes, persistently high unit emission reduction costs, and significant risks to the power system’s supply security.

Gradually introducing auctions and tightening quotas represents the optimal path for achieving sustainable carbon market operation and energy structure optimization. In the smooth scenario, power enterprises gradually increased the quota reduction ratio and auction ratio, enabling the phased implementation of low-carbon technology renovations and investments in non-fossil energy under manageable carbon constraints. This approach achieved a coordinated optimization of market stability, cost control, and emission reduction effectiveness. In this scenario, the carbon price trend remained stable—1,678 yuan/ton in the primary market and 2,182 yuan/ton in the secondary market by 2060—with a cumulative emission reduction of 377.7 billion tons. The unit emission reduction cost was significantly lower than in the synchronous scenario, effectively mitigating the risks of carbon price collapse and power supply interruptions. Therefore, the compromise solution of smooth advancement ensures energy security and economic affordability while balancing long-term carbon market stability with deep emission reductions. The EU ETS serves as a conceptual reference, highlighting the importance of sequencing quota tightening and auction expansion, with implementation tailored to China’s conditions.

### Policy implications

Based on the aforementioned research conclusions, we propose the following suggestions:

For the government, comprehensively assessing the intensity and cost of emission reduction, gradually introducing auctions, and tightening carbon quotas can prevent auctions from causing excessive carbon pressure. This approach allows enterprises sufficient time to adjust, creates a stable market environment, and provides an effective carbon price signal, thereby better promoting emission reduction in the power industry. At the same time, policymakers should strengthen the coordination between carbon market reform and power system transformation. In practice, the effectiveness of stronger carbon pricing signals depends on complementary policies such as renewable integration support, energy storage deployment, demand response, and grid flexibility enhancement. In addition, auction revenues can be recycled to support low-carbon technology upgrading and renewable energy investment, thereby improving both the efficiency and acceptability of carbon market reform.

For power enterprises, it is essential to accelerate the implementation of low-carbon technological renovations. While ensuring a stable electricity supply, they should expedite the decommissioning of fossil energy generating units and increase investment in non-fossil energy units. They should actively participate in carbon market transactions to avoid tight carbon quotas at the end of the compliance period, which could result in increased carbon costs.

For the power grid, it is essential to ensure a stable power supply, guide enterprises in establishing a reasonable configuration of power generation sources, set a minimum installed capacity threshold for thermal power units, and maintain sufficient power generation margins to prevent grid collapse caused by the unstable output of new energy sources. Simultaneously, it is also essential to accelerate the process of synergy in the electric and carbon market, establish a complete management mechanism for the auction income of the carbon market, guide the carbon market income subsidies to tilt toward renewable energy, strengthen the role of the carbon market in promoting the development of renewable energy. More broadly, better coordination among the electricity market, the carbon market, and related environmental policy instruments—such as green certificate trading—would further improve the pass-through of carbon costs and the consistency of long-term decarbonization incentives.

In line with COP30’s emphasis on deep, verifiable, and interconnected carbon markets, this study offers actionable insights for international market design. By demonstrating how phased auction introduction and dynamic quota reduction stabilize prices, guide low-carbon investments, and maintain system reliability, our findings provide transferable lessons for emerging and established carbon markets worldwide, supporting the implementation of high-integrity, coordinated, and effective emission-reduction mechanisms.

### Limitations of the study

While this study provides a system-level analysis of carbon market design using a multi-agent simulation framework, two limitations should be noted. First, the electricity market is modeled as an energy-only market, without explicitly representing regional heterogeneity, transmission constraints, renewable integration limits, capacity mechanisms, or ancillary services; incorporating these features in future work would improve the assessment of system flexibility, reliability, and carbon cost pass-through. Second, firm behavior is simplified as cost-minimization within a multi-agent framework, abstracting from behavioral heterogeneity such as risk aversion, financing constraints, and technological lock-in; future research could integrate empirically grounded behavioral rules to enhance realism. Addressing these aspects would further strengthen the linkage between stylized simulation and real-world policy design.

## Resource availability

### Lead contact

Further information and requests for resources and reagents should be directed to and will be fulfilled by the lead contact, Lu-Tao ZHAO (ltzhao@bit.edu.cn).

### Materials availability

This study did not generate new unique reagents.

### Data and code availability

• All data utilized in this study are declared in STAR Methods.
• This article reports the original code, which has been made publicly available via DOI at https://doi.org/10.5281/zenodo.20092191.
• Any additional information required to reanalyze the data reported in this paper is available from the lead contact upon request.

## Acknowledgments

This work was supported by the 10.13039/501100001809National Natural Science Foundation of China (No. 72271028), the Fundamental Research Funds for the Central Universities, and the Natural Science Foundation of Hebei Province (No. G2025105001).

## Author contributions

Z.-Y.L.: conceptualization, data curation, formal analysis, methodology, software, and writing – original draft; L.-T.Z.: conceptualization, funding acquisition, project administration, supervision, validation, and writing – review and editing; Z.Q.: investigation, validation, and writing – review and editing; Z.-Y.C.: conceptualization, validation, and writing – review and editing; R.-X.Q.: conceptualization, validation, and writing – review and editing; X.-Y.A.: investigation, validation, and writing – review and editing; D.-S.W.: investigation, validation, and writing – review and editing.

## Declaration of interests

The authors declare no competing interests.

## STAR★Methods

### Key resources table

**Table:** 

REAGENT or RESOURCE	SOURCE	IDENTIFIER
**Software and algorithms**		

Python version 3.13.0	Python Software Foundation	https://www.python.org/
Mesa version 3.0.0b2	Mesa Team	https://github.com/mesa/mesa
ECMAS-v1	This paper	https://doi.org/10.5281/zenodo.20092191
Origin 2024	OriginLab Corporation	https://www.originlab.com/origin

### Method details

#### Model framework

**Figure 6:**
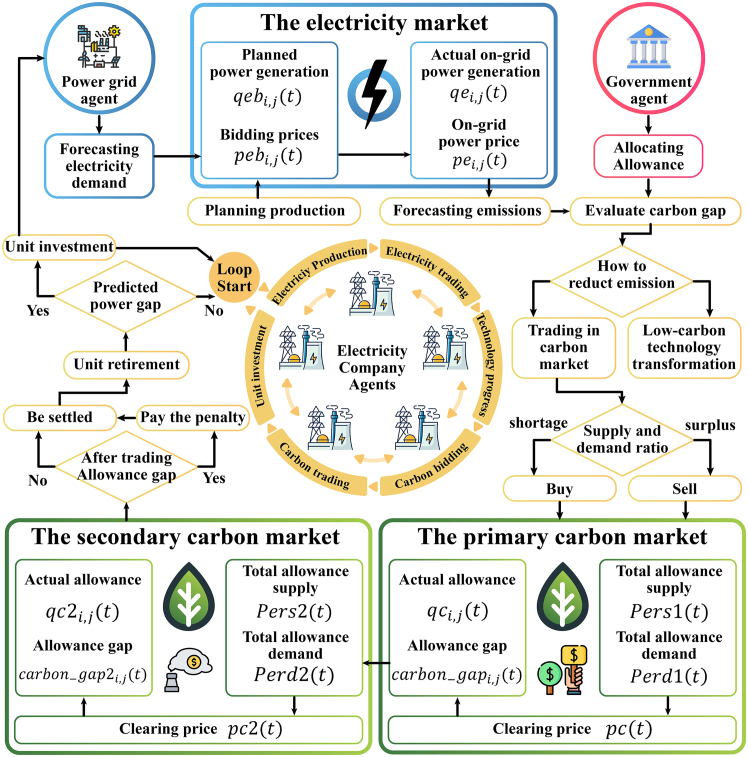
Framework of the ECMAS

Based on the theory of Complex Adaptive Systems (CAS) and agent-based model (ABM), we aimed to simulate the behavioral rules and interactions of various entities within the complex environment of the electricity and carbon markets from a bottom-up perspective. This approach facilitates the development of low-carbon transition pathways for the electricity industry under different policy scenarios. Considering the current state of China’s electricity industry and national carbon market, we constructed a multi-agent simulation model comprising six modules: electricity production, electricity trading, low-carbon technology transformation, carbon allowance allocation, carbon allowance trading, and unit investment and retirement. We defined three types of entities: electricity companies, government and power grid, which interact through three markets: the electricity market, the primary carbon market, and the secondary carbon market. Notably, the electricity market operates as a competitive market. The Framework of ECMAS is shown in Figure 6.

#### Module details

We set 2020 as the base year. With an annual time frequency, the low-carbon transition pathway for China’s electricity industry from 2020 to 2060 can be developed through an autoregressive process. The key results include, but are not limited to, carbon prices, carbon emissions, electricity generation structure, and emission reduction costs. The main parameters and variables of the model are shown in Supplemental information document S1. We utilized the Mesa library, designed for multi-agent modeling, for the simulation. The operating environment is Python 3.9.13.

##### Electricity production module

First, referring to literature,^57^ the annual electricity demand is updated as shown in Equation 1. We assumed that China’s electricity demand is driven by GDP and electricity prices.

(Equation 1) $$D\left(t\right)=D\left(t-1\right)\cdot \left[1+\alpha_{gdp}\left(t-1\right)\cdot \rho_{gdp}\right]\cdot \left[1+\alpha_{pe}\left(t-1\right)\cdot \rho_{pe}\right]$$

Then, each power generation enterprise submits its planned power generation and power generation bid price to the grid operator according to its maximum power generation capacity. The planned power generation is determined by installed capacity and annual utilization hours, as shown in Equation 2.

(Equation 2) $${qeb}_{i,j}\left(t\right)={capacity}_{i,j}\left(t\right)\cdot {ehour}_{j}$$

Bidding prices are based on the principle of cost plus, which enables the power generation enterprises to achieve its expected profit rate on the basis of the cost of each type of unit, as shown in Equation 3.

(Equation 3) $$peb_{i,j}\left(t\right)=\left[fc_{i,j}\left(t\right)\cdot qeb_{i,j}\left(t\right)+omc_{i,j}\left(t\right)\cdot qeb_{i,j}\left(t\right)+tech_{i,j}\left(t\right)+\left(car_{-}in_{i,j}\left(t\right)-Be_{j}\left(t\right)\right)\cdot qeb_{i,j}\left(t\right)\cdot pc2\left(t-1\right)\right]\cdot \left(1+mar_{i}\cdot ave_{-}mar\right)/qeb_{i,j}\left(t\right)$$

##### Electricity trading module

In this study, the electricity market is modeled as an energy-only market (EOM), focusing on the electricity energy market where generation units participate through marginal cost-based bidding and are dispatched according to economic dispatch principles under carbon pricing. Capacity remuneration mechanisms and ancillary service markets are not explicitly represented. This modeling choice allows us to isolate the effects of carbon pricing and allowance allocation on dispatch and investment decisions, while avoiding additional complexity from multi-market interactions.

In the process of trading in the electricity market, the grid operator determines the actual generation dispatch of each power plant based on the submitted power supply and demand situation and the priority of power dispatch. According to the guiding principles of the “Basic Rules for Medium and Long-term Electricity Trading”, the priority order for entering the electricity market is gas power, adjustable hydropower, nuclear power, non-adjustable hydropower, wind power, and photovoltaic power.

Therefore, the grid operator prioritizes the on-grid power generation of gas power, hydropower, nuclear power, wind power, and photovoltaic units, meaning that the actual on-grid power generation quantity of these five types of units is their submitted planned generation quantity *qeb*_*i*,*j*_, while the residual power demand is met by coal power units. For coal power units, the grid operator allocates their on-grid power generation quantity based on the bidding price, as shown in Equation 4, the higher the bidding price submitted, the lower the winning bid quantity.

(Equation 4) $$qe_{i,j}\left(t\right)=qeb_{i,j}\left(t\right)\cdot \frac{D\left(t\right)}{\sum \sum qeb_{i,j}\left(t\right)}\cdot \frac{bid_{-}ave\left(t\right)}{peb_{i,j}\left(t\right)}$$

The average on-grid power price *e*_-_*price*(*t*) in the electricity market is determined by the overall power supply and demand situation and the average electricity bidding price *bid*_-_*ave*(*t*) of each power generation enterprise, as shown in Equations 5 and 6.

(Equation 5) $$e_{-}price\left(t\right)=bid_{-}ave\left(t\right)\cdot e^{\theta \cdot \left(\frac{D\left(t\right)}{\sum \sum qeb_{i,j}\left(t\right)}-1\right)}$$

(Equation 6) $$bid_{-}ave\left(t\right)=\frac{\sum \sum qeb_{i,j}\left(t\right)\cdot peb_{i,j}\left(t\right)}{\sum \sum qeb_{i,j}\left(t\right)}$$

The on-grid power price *pe*_*i*,*j*_(*t*) is determined based on the average on-grid power price and its own electricity bidding price, as shown in (Equation 5), (Equation 6).

(Equation 7) $$pe_{i,j}\left(t\right)=e_{-}price\left(t\right)\cdot \frac{peb_{i,j}\left(t\right)}{bid_{-}ave\left(t\right)}$$

##### Low-carbon technology transformation module

First, power enterprises calculate the carbon emissions generated by thermal power generation based on the current carbon emission intensity of the unit. Then compare it with the carbon allowance allocated for free by the government. Any excess emissions are recorded as the allowance difference *carbon*_*gap*_*i*,*j*_(*t*), as shown in (Equation 5), (Equation 6).

(Equation 8) $$carbon_{-}gap_{i,j}\left(t\right)=qe_{i,j}\left(t\right)\cdot car_{-}in_{i,j}\left(t\right)-carbon_{-}free_{i,j}\left(t\right)$$

When *carbon*_*gap*_*i*,*j*_(*t*) > 0, which means that the estimated carbon emissions of the enterprise exceed the allowance allocated for free distribution, the power generation enterprise will compare the unit reduction costs of low-carbon technology transformations with the estimated carbon price. Based on the current status of the units that have undergone transformation, it will calculate the remaining reduction potential for each technology and prioritize transformations in sequence, starting with those technologies whose unit reduction costs are lower than the carbon price, as shown in (Equation 5), (Equation 6).

(Equation 9) $$Tech_{i,te}\left(t\right)=\left\{ \begin{array}{cc} 0, & CCE_{te}>pc^{\prime }\left(t\right)\\ carbon_{-}gap_{i,j}\left(t\right), & Tech_{-}surp_{i,te}\left(t\right)\geq carbon_{-}gap_{i,j}\left(t\right)\\ Tech_{-}surp_{i,te}, & Tech_{-}surp_{i,te}<carbon_{-}gap_{i,j}\left(t\right) \end{array}\right.$$

In reality, power generation enterprises tend to prefer higher capital returns and shorter investment payback periods.^58^ Therefore, it is assumed that all emission reduction technologies have the same discount rate and investment return rate. These assumptions are adopted to isolate the effects of carbon market mechanisms from behavioral heterogeneity, allowing us to focus on the core drivers of investment decisions under carbon pricing. While these assumptions simplify the complex decision-making process, they provide a baseline for understanding the aggregate impact of carbon market design. The selected low-carbon transformation technology for coal-fired power will be detailed in section 4.3. The unit emission reduction cost of low-carbon technology is shown in (Equation 5), (Equation 6).

(Equation 10) $$CCE_{te}=\frac{I_{te}\cdot \frac{d}{\left(1-{\left(1+d\right)}^{-n}\right)}}{CS_{te}}$$

##### Carbon allowance allocation module

The allocation of allowances obtained by emission enterprises from administrative departments can be divided into “free allocation” and “paid allocation” based on whether costs need to be incurred. Typically, these two allocation methods are used in combination according to a certain ratio.

In the free allocation period, the reduction targets can be measured through two approaches: mass-based and rate-based. For estimating carbon allowance demand, there are the “grandfathering approach” and the “benchmarking approach.” In China’s national carbon market, the emission standards based on the highest reduction efficiency among similar equipment are used as benchmarks, and after certain adjustments, the actual allowances that enterprises can be allocated are determined. We have also adopted this mass-based benchmarking method for calculations.

First, the government agent calculates the carbon emission intensity benchmark value for type *j* units in thermal power generation each year according to (Equation 5), (Equation 6).

(Equation 11) $$Be_{j}\left(t\right)=\frac{\sum qe_{i,j}\left(t\right)\cdot car_{-}in_{i,j}\left(t\right)}{\sum qe_{i,j}\left(t\right)}$$

Then, the free allowances obtained by the electricity companies are calculated according to (Equation 5), (Equation 6). where, *μ* is the auction ratio, and *R* is the allowance reduction ratio.

(Equation 12) $$carbon_{-}free_{i,j}\left(t\right)=qe_{i,j}\left(t\right)\cdot Be_{j}\left(t\right)\cdot \left(1-R\right)\cdot \left(1-\mu \right)$$

In the paid allocation period, the auction mechanism is the mainstream. Emission enterprises participate in auctions on the exchange to obtain carbon allowances, and the funds raised from the auctions are redistributed by government to carbon market construction and emission reduction action. As shown in Table 3, standard auctions can be divided into two types: static (sealed) auctions and dynamic (clock) auctions.^59^

**Table 3:** Standard auction formats

Static (sealed) auction	Dynamic (clock) auction
Uniform-price auction	ascending auction (English auction)
Discriminatory-price auction	descending auction (Dutch auction)
Second-price sealed auction (Vickrey auction)	–

Static auctions can be further classified into three types based on the clearing price: uniform price auction, where the clearing price is the equilibrium price; discriminatory price auction, where the clearing price is the bidder’s own bid; and second-price auction, where the clearing price is the opportunity cost of the carbon allowance.

Dynamic auctions can be further classified into ascending auctions and descending auctions based on the price adjustment method. The auctioneer provides a price in each round. Bidders adjust their bid quantities based on the price. The auctioneer raises or lowers the price in each round until the total bid quantity equals the quantity of the auctioned item.

Currently, uniform price auctions are widely used in the carbon market around the world. Therefore, we assumed that all enterprises participating in the primary carbon market auctions acquire compensated allowances at the same price.

Consider that some power generation enterprises may reduce emissions by adopting low-carbon technologies as mentioned in section 2.3. The carbon allowance gap for each power generation enterprise will be updated as shown in (Equation 5), (Equation 6).

(Equation 13) $$carbon_{-}gap_{i,j}\left(t\right)=qe_{i,j}\left(t\right)\cdot car_{-}in_{i,j}\left(t\right)-carbon_{-}free_{i,j}\left(t\right)-Tech_{i,te}\left(t\right)$$

If *carbon*_*gap*_*i*,*j*_(*t*) > 0, meaning that the actual carbon emissions of the power generation enterprise exceed the free allocation, the enterprise first acquires paid allowances through auctions. In the primary carbon market, the supplier of allowances is the government, and the total supply is the portion of the total allowance divided according to the auction ratio *μ*, as shown in (Equation 5), (Equation 6);

(Equation 14) $$Pers1\left(t\right)=carbon_{-}auc\left(t\right)=\sum_{i}\;\sum_{j}\;qe_{i,j}\left(t\right)\cdot Be_{j}\left(t\right)\cdot \left(1-R\right)\cdot \mu$$

the demander of allowances is power generation companies that are experiencing an allowance shortage. The total demand is the sum of the allowance shortages of all power generation enterprises participating in the primary market transactions, as shown in (Equation 5), (Equation 6).

(Equation 15) $$Perd1\left(t\right)=\sum_{i}\;\sum_{j}\;carbon_{-}gap_{i,j}\left(t\right),carbon_{-}gap_{i,j}\left(t\right)>0$$

The carbon price in the primary market for this period is determined by the carbon price in the secondary market from the previous period and the current market supply and demand relationship, as shown in (Equation 5), (Equation 6).

(Equation 16) $$pc\left(t\right)=pc2\left(t-1\right)\times e^{\lambda_{1}\cdot \left(Perd1/Pers1-1\right)}$$

The allowance purchased by power generation enterprises in the primary carbon market is as shown in (Equation 5), (Equation 6).

(Equation 17) $$qc_{i,j}\left(t\right)=\frac{carbon_{-}gap_{i,j}\left(t\right)}{Pers1}\cdot Perd1$$

##### Carbon allowance trading module

After trading in the primary carbon market, power generation enterprises update their allowance gaps once again, as shown in (Equation 5), (Equation 6). If a company has a surplus of allowances (*carbon*_-_*gap*_*i*,*j*_(*t*)) or still faces a shortage (*carbon*_-_*gap*_*i*,*j*_(*t*) > 0), they will trade in the secondary carbon market.

$$carbon_{-}gap_{i,j}\left(t\right)=qe_{i,j}\left(t\right)\cdot car_{-}in_{i,j}\left(t\right)-carbon_{-}free_{i,j}\left(t\right)$$

(Equation 18) $$-Tech_{i,te}-qc_{i}\left(t\right)$$

In the secondary carbon market, the allowance suppliers are power generation enterprises that have an allowance surplus. The total supply is the sum of the allowance surplus of these power generation enterprises, as shown in (Equation 5), (Equation 6);

(Equation 19) $$Pers2\left(t\right)=\sum_{i}\;\sum_{j}\;{\;carbon\_gap\;}_{i,j}\left(t\right),{\;carbon\_gap\;}_{i,j}\left(t\right)<0$$

The allowance demanders are power generation enterprises that still have an allowance shortage. The total demand is the sum of the allowance shortage of these power generation enterprises, as shown in (Equation 5), (Equation 6).

(Equation 20) $$Perd2\left(t\right)=\sum_{i}\;\sum_{j}\;{\;carbon\_gap\;}_{i,j}\left(t\right),{\;carbon\_gap\;}_{i,j}\left(t\right)>0$$

The price in the secondary carbon market is determined by the market supply and demand situation based on the current auction price, as shown in (Equation 5), (Equation 6). At the same time, the government will regulate the carbon price in the secondary market to maintain market stability. According to the Shanghai Environment and Energy Exchange, block trading prices are allowed to fluctuate within ±30% of the previous day’s closing price, while listed agreement trading is subject to a narrower ±10% band.^60^ Referring to the practices in ref.^61^ and given that our model focuses on aggregate market behavior and large-volume transactions, the carbon price in the secondary market will fluctuate within ±30% of the carbon price in the primary market.

(Equation 21) $$pc2\left(t\right)=pc\left(t\right)\times e^{\lambda_{2}\cdot \left(\;Perd\;2/\;Pers\;2-1\right)}$$

The trading volume of each power generation enterprise in the secondary carbon market will also be determined by the market supply and demand situation.^61^

When the market is in short supply, i.e., a seller’s market, all selling companies will sell their entire allowances, while buying companies can only purchase a corresponding proportion of the allowances, with the trading volume calculated as shown in (Equation 5), (Equation 6);

(Equation 22) $$qc2_{i,j}\left(t\right)=\left\{ \begin{array}{cc} \frac{carbon_{-}gap_{i,j}}{Perd2}\cdot Pers2, & carbon_{-}gap_{i,j}>0\\ carbon_{-}gap_{i,j}, & carbon_{-}gap_{i,j}<0 \end{array}\right.$$

When the market is oversupplied, i.e., a buyer’s market, all buying companies can purchase the required allowances, while selling companies can only sell a corresponding proportion of the allowances, with the trading volume calculated as shown in (Equation 5), (Equation 6).

(Equation 23) $$qc2_{i,j}\left(t\right)=\left\{ \begin{array}{cc} carbon_{-}gap_{i,j}, & carbon_{-}gap_{i,j}>0\\ \frac{carbon_{-}gap_{i,j}}{Pers2}\cdot Perd2, & carbon_{-}gap_{i,j}<0 \end{array}\right.$$

After all carbon trading is completed, power generation enterprises will fulfill their allowance settlement obligations, and the government will calculate the actual carbon emissions of the power generation enterprises, imposing penalty on the portion that exceeds their allowances, as shown in (Equation 5), (Equation 6). Where, *ξ* is the penalty rate.

(Equation 24) $$Penalty_{i,j}\left(t\right)=\xi \cdot pc2\left(t\right)\cdot [qe_{i,j}\left(t\right)\cdot car_{in_{i,j}}\left(t\right)-carbon_{free_{i,j}}\left(t\right)-Tech_{i,te}-qc_{i,j}\left(t\right)-qc2_{i,j}\left(t\right)]$$

##### Unit investment and retirement module

First, the power generation enterprises will predict the electricity demand for the next year using regression methods based on the electricity demand situation over the past five years. When the ratio of the next year’s electricity demand to the available electricity supply exceeds the investment threshold *ε*_*i*_, which satisfies (Equation 5), (Equation 6), it indicates that there may be a shortage in future electricity supply, prompting the companies to invest in new generating units.^62^ The types of generating units that power generation enterprises can choose to invest in include six categories: coal power, gas power, hydro power, wind power, photovoltaic power, and nuclear power.

(Equation 25) $$\frac{D_{-}pred\left(t+1\right)}{qe_{-}now\left(t\right)+qe_{-}new\left(t\right)-qe_{-}retire\left(t\right)}>\varepsilon_{i}$$

Next, when the power generation enterprises decide to invest in new units, they will determine the design capacity of the new units based on their forecasted electricity shortage and their current market share in the electricity market, as shown in (Equation 5), (Equation 6).^34^^,^^62^

(Equation 26) $$qe_{-}inv_{i,j}\left(t\right)=\left(\frac{D\_pred\left(t\right)}{\varepsilon_{i}}-qe_{-}now\left(t\right)+qe_{-}new\left(t\right)-qe_{-}retire\left(t\right)\right)\cdot \frac{qe_{i}}{\sum qe_{i}}$$

Then, the power generation enterprises will make investment decisions regarding the installed capacity of different types of units based on the principle of economic efficiency, considering the economic factors of each power generation technology. The investment return rate is the ratio of the net present value of the estimated total revenue over the entire life cycle of the new unit to the initial capital investment of the power plant, as shown in Equations 27 and 28.

(Equation 27) $$\begin{array}{c} Profit_{i,j}\left(t\right)=pe_{i,j}\left(t\right)\cdot qe_{-}inv_{i,j}\left(t\right)-pc2\left(t\right)\cdot \left(qe_{-}inv_{i,j}\left(t\right)-Be_{j}\left(t\right)\right)\\ \;\cdot qe_{-}inv_{i,j}\left(t\right)-fc_{i,j}\left(t\right)-omc_{i,j}\left(t\right) \end{array}$$

(Equation 28) $$\begin{array}{c} W_{i,j}\left(t\right)=\frac{\sum_{t^{\prime }=t}^{life_{i,j}+t}\;\left\{\frac{Profit_{i,j}\left(t\right)}{{\left(1+d\right)}^{t^{\prime }-t}}\right\}}{invest_{-}\;\cos\;t_{i,j}}-1+restrate_{i,j} \end{array}$$

The economic factor for each power source is the expected discounted rate of return on investment for that unit, expressed as a proportion of all types of units, as shown in (Equation 27), (Equation 28).

(Equation 29) $$eco_{-}fac_{i,j}\left(t\right)=\frac{w_{i,j}\left(t\right)}{\sum_{j}\;w_{i,j}\left(t\right)}$$

At the same time, hydro, wind, photovoltaic, and nuclear power generation heavily depend on resources in the natural environment. Therefore, there is a maximum resource constraint on the total installed capacity of various power sources, as shown in (Equation 27), (Equation 28).

(Equation 30) $$\sum_{i}\;capacity_{i,j}\leq RC_{j}$$

Additionally, we assumed that the investment costs of each power generation technology experience endogenous technological progress, which is simulated through a learning-by-doing model to reflect the process of endogenous technological advancement, as shown in (Equation 27), (Equation 28). The selection of learning rates is based on a synthesis of empirical studies. Rubin et al. (2015) show that learning rates vary significantly across technologies and studies, often spanning a wide range due to differences in data, time periods, and model specifications.^63^ In line with this evidence, this study assigns differentiated learning rates according to technology maturity: lower values (e.g., 0.5%–1%) are used for mature technologies such as thermal and nuclear power, while higher values (around 15%–20%) are applied to renewable technologies such as wind and solar power (detailed in Appendix A). These values fall within the representative ranges reported in the literature.

(Equation 31) $$invest_{-}\;\cos\;t_{j}\left(t\right)=invest_{-}\;\cos\;t_{j}\left(t_{0}\right){\left(\frac{\sum_{i}\;capacity_{j}\left(t\right)}{\sum_{i}\;capacity_{j}\left(t_{0}\right)}\right)}^{-\sigma_{j}}$$

Finally, when an old power plant reaches the end of its lifespan or incurs operational losses for five consecutive years, the power generation enterprise will retire the plant and recover its residual value.

#### Parameter settings and data sources

##### Economic Parameters

The main economic parameters of the model are shown in Table S1. The GDP growth rate is based on the medium-speed scenario forecast from ref.^64^, with an average growth rate of 5.025% from 2021 to 2040. The equilibrium ratio coefficient of the primary carbon market is estimated using empirical data from the Guangdong carbon market’s auction transactions from 2013 to 2019, as compiled in ref.^65^ The carbon market penalty rate is referenced from the settings in ref.^61^ The remaining parameters are primarily based on the multi-agent simulation model of the electricity industry constructed in ref.^37^^,^^57^ The baseline carbon price is set based on the actual operation of the national carbon market in 2021, where the annual average closing price for each trading day in the national carbon market was approximately 50 yuan/ton.

##### Power industry parameters

We focus on six different types of power sources. The key parameters for each source include cost, technical specifications, and carbon emission intensity, among others. Detailed settings and data sources are provided in Table S1. Specifically, the average annual operating hours for power generation are calculated by averaging the equipment utilization hours from the past five years, as reported in the “China Electric Power Statistical Yearbook.” The carbon emission intensity for coal-fired units is determined by multiplying the benchmark coal consumption for power generation of each unit, as outlined in the “Benchmark and Baseline Levels for Key Areas of Clean and Efficient Utilization of Coal (2022 Edition),” by the proportion of its installed capacity and then by the emission factor. For gas-fired units, the carbon emission intensity is based on the benchmark value from the “Implementation Plan for the Total Amount Setting and Allocation of National Carbon Emission Trading Allowances for 2021 and 2022 (Power Generation Industry)” for the year 2021.

##### Low-carbon technology transformation parameters

The energy-saving renovation project for coal-fired power units mainly includes energy-saving and low-carbon renovation technologies in four aspects: the main boiler, boiler auxiliary machines, the main steam turbine, and steam turbine auxiliary machines. Referring to the “National Key Energy-Saving and Low-Carbon Technology Promotion Catalog” and literature,^66^^,^^67^ we primarily selects six types of low-carbon renovation technologies for coal-fired power. The specific technical parameters are shown in Table S3.

#### Model configuration

According to data from the National Carbon Market Information Network, there are a total of 2,261 key emission units in the power industry included in the national carbon market by 2025. Therefore, this model initializes with 2,261 power enterprise entities. The installed capacity of each power enterprise at the baseline period is averaged based on the national installed capacity shown in Table S2.

Recognizing the variability in efficiency levels among coal-fired power units, we posit that there exists heterogeneity in carbon emission intensity across different power enterprises. As detailed in Table 4, coal-fired power units are classified into three categories: Benchmark level, Baseline level, and Lagging level. The first two categories are derived from the criteria established in the “Benchmark and Baseline Levels for Key Areas of Clean and Efficient Utilization of Coal (2022 Edition),” wherein the benchmark level represents the average value of the top 20% of thermal power efficiency indicators across various unit types. The carbon emission intensity for both benchmark and baseline level units are computed by multiplying the respective coal consumption per unit of electricity generated by their current installed capacity proportion, followed by the application of the emission factor.

**Table 4:** Heterogeneity settings of agents

Level	Carbon emission intensity (tCO_2_/MWh)	Proportion
Benchmark	0.8177	20%
Baseline	0.8729	60%
Lagging	0.9303	20%

Furthermore, we assume that the weighted average carbon emission intensity of these three categories of units reflects the equilibrium carbon emission intensity for coal-fired power units, as articulated in the “Implementation Plan for the Total Amount Setting and Allocation of National Carbon Emission Trading Allowances for 2021 and 2022 (Power Generation Industry).” Based on the estimated carbon emission intensity of this plan, we have set the parameters for the three types of units as shown in Table 4.

#### Model calibration

After constructing the multi-agent simulation model for the power industry and setting the parameters, the model was further validated. By inputting power industry data from 2022 as the base year, the operational status of the power industry in 2023 was simulated, and then compared with actual data to verify the model’s effectiveness, as shown in Table 5. The relative errors of all indicators were within 5%, indicating that the multi-agent simulation model for the power industry constructed in this paper is reasonably effective to a certain extent.

**Table 5:** Model calibration results

Indicators	Unit	2023 Forecast value	2023 Actual value	Relative error
Total power generation	100 million kWh	91,144	94,393	−3.44%
Thermal power generation	100 million kWh	59,851	62,657	−4.47%
Non-fossil energy power generation	100 million kWh	30,293	29,471	2.78%
Thermal power installed capacity	10,000 kW	133,143	139,032	−4.23%
Non-fossil energy installed capacity	10,000 kW	147,843	153,225	−3.51%
