# Supplementary material for: Gradual introduction of carbon allowance auctions facilitates sustainable emission reductions in the power sector
Source: iScience. 2026 Jun 8;29(6):116241. doi: 10.1016/j.isci.2026.116241 (PMC13264093; doi:10.1016/j.isci.2026.116241)
Supplement: Document S1. Tables S1–S5 [file mmc1.pdf]

iScience, Volume *29*

Supplemental information

Gradual introduction of carbon allowance auctions facilitates sustainable emission reductions in the power sector

Zhao-Yuan Li, Lu-Tao Zhao, Zhi Qu, Zhe-Yi Chen, Rui-Xiang Qiu, Xing-Yu An, and Dai-Song Wang

Document S1 Main Parameters and variables

Table S1 Main parameters and exogenous variables

Variables	Definition	Unit	Value	Data sources
α_{gdp}	The growth rate of GDP	%	0.05025	1
ρ_{gdp}	GDP elasticity coefficient of electricity demand	-	0.8	2
ρ_{eprice}	Electricity price elasticity coefficient of electricity demand	-	-0.1	2
mar_i	The ratio of profit margins of electricity providers to the average profit margins of electric power industry	-	1	2
ave_mar	The average profit margin for the power industry		0.1	2
θ	Power market equilibrium ratio coefficient		0.001	2
ε_i	Power unit investment threshold	-	0.9436	3
d	Discount rate	-	0.08	3
λ_1	Primary carbon market equilibrium ratio coefficient	-	0.12	4
λ_2	Secondary carbon market equilibrium ratio coefficient	-	0.05	2
ξ	Carbon market penalty rate	-	3	5
$pc2_{t0}$	Base period carbon price	Yuan/ton	48	Wind

Table S2 technical parameters

Parameters	Definition	Unit	Thermal power	Gas power	Hydro power	Wind power	Solar power	Nuclear power	Data sources
$invest\_cost_j$	Investment costs	Yuan/kW	4569	4569	4569	4569	4569	4569	[6,7]
σ_j	Learning rates for electricity technologies	/	0.01	0.008	0.005	0.16	0.16	0.008	[8]
om_cost_j	Operational costs	Yuan/kWh	0.0131	0.0131	0.0131	0.0131	0.0131	0.0131	[9]
$fuel\_cost_j$	Unit fuel costs	Yuan/kWh	0.241	0.563	/	/	/	0.041	[3]
eco_fact_j	Experience factor of Technological Progress	/	0.03	0.03	0.03	0.03	0.03	0.03	[10,11]
$capcons_j$	Ratio of remaining value	/	0.05	0.05	0.05	0.05	0.05	0.05	[3]
$ehour_j$	Annual utilization hours	hour	4300	3200	3700	1700	1200	7200	"China Electric Power Statistical Yearbook"
$tot\_cap_j(0)$	Installed capacity in the base period	10^4kW	107912	107912	107912	107912	107912	107912	"China Electric Power Statistical Yearbook"
cap_cons_j	Installed capacity constrains	10^4kW	/	/	54000	54000	54000	54000	[9,12]

Table S3 Low-carbon technology transformation parameters

Technology number	Technology type	Investment Amount (Million Yuan/Megawatt)	Emission Reduction Potential (Tons/Megawatt per Year)	Unit Emission Reduction Cost (Yuan/Ton)
T1	Boiler Combustion Temperature Control and Performance Optimization System	0.82	18	42.68
T2	Condenser spiral coil descaling device technology	3	54	52.04
T3	Power Plant Boiler Intelligent Ash Cleaning Optimization and Online Coking Early Warning System	0.18	3	56.21
T4	Electrostatic Precipitator Energy Saving and Efficiency Improvement Control Technology	0.9	12	70.26
T5	Quasi-stable dc dust collector power supply energy saving technology	2.4	21	107.06
T6	Turbine flow part modernization retrofit	12.81	35	342.86

Table S4 Main subscript and definition

Subscript	Definition	Range
i	the ith enterprise	1-2262
j	the jth power source	1-6
te	the teth power generation technology	1-6

Table S5 Main Endogenous variables

Variables	Definition
$D(t)$	Annual electricity demand
$e\_price(t)$	Average on-grid power price
$qeb_{i,j}(t)$	Planned power generation
$capacity_{i,j}(t)$	Installed capacity
$peb_{i,j}(t)$	Bidding prices
$fc_{i,j}(t)$	Fuel costs
$omc_{i,j}(t)$	Operating and maintenance costs
$tech_{i,j}(t)$	Low carbon technology renovation costs
$car\_in_{i,j}(t)$	Carbon intensity
$Be_j(t)$	Carbon intensity at baseline
$qe_{i,j}(t)$	Actual on-grid power generation
$pe_{i,j}(t)$	On-grid power price
$bid\_ave(t)$	The average electricity bidding price
$carbon\_gap_{i,j}(t)$	Carbon quota supply and demand gap
$carbon\_free_{i,j}(t)$	Carbon quota allocated for free
$Tech_{i,te}(t)$	Technical emissions reduction achieved after adopting low-carbon technologies
$Tech\_surp_{i,te}(t)$	Remaining emissions reduction potential of units that have not undergone low-carbon technology transformations.
CCE_{te}	Unit emission reduction cost
I_{te}	The investment cost of the low-carbon technology

CS_{te}	
$Pers1(t)$	Total supply for quotas in the primary carbon market
$Perd1(t)$	Total demand for quotas in the primary carbon market
$pc(t)$	Clearing price of carbon quotas in the primary carbon market
$qc_{i,j}(t)$	Quantity of carbon quotas in the primary carbon market
$Pers2(t)$	Total supply for quotas in the secondary carbon market
$Perd2(t)$	Total demand for quotas in the secondary carbon market
$pc2(t)$	Clearing price of carbon quotas in the secondary carbon market
$qc2_{i,j}(t)$	Quantity of carbon quotas in the secondary carbon market
$Penalty_{i,j}(t)$	Carbon compliance penalty
$D\_pred(t+1)$	The estimated total electricity demand for the next year
$qe\_now(t)$	The available electricity supply from all existing generating units
$qe\_new(t)$	The available electricity supply from newly invested generating units that will be put into production next year
$qe\_retire(t)$	The available electricity supply from units expected to be retired next year
$qe\_inv_{i,j}(t)$	The design capacity of the new units
$W_{i,j}(t)$	Investment return rate
$eco\_fac_{i,j}(t)$	The economic factor for each power source
RC_j	Maximum resource constraint on the total installed capacity of various power sources
$invest\_cost_j(t)$	Investment costs of each power generation technology

Reference

1. Wei Y., Yu B., Tang B., Liu L., Liao H., Chen J., Sun F., An R., Wu Y., Tan J., et al. (2022). Roadmap for Achieving China's Carbon Peak and Carbon Neutrality Pathway. Journal of Beijing Institute of Technology(Soci al Sciences Edition) *24*, 13–26. https://doi.org/10.15918/j.jbitss1009-3370.20 22.1165.
2. Cong, R.-G., and Wei, Y.-M. (2010). Potential impact of (CET) carbon em issions trading on China's power sector: A perspective from different allow ance allocation options. Energy *35*, 3921–3931. https://doi.org/10.1016/j.ener gy.2010.06.013.
3. Chen, H., Wang, C., Cai, W., and Wang, J. (2018). Simulating the impact of investment preference on low-carbon transition in power sector. Applied Energy *217*, 440–455. https://doi.org/10.1016/j.apenergy.2018.02.152.
4. Wang, W., Zhao, X., Zhang, Q., Fu, C., and Xie, P. (2022). Auction mech anism design of the Chinese national carbon market for carbon neutralizati on. Chinese Journal of Population, Resources and Environment *20*, 115–12 4. https://doi.org/10.1016/j.cjpre.2022.06.002.
5. Tang, L., Wu, J., Yu, L., and Bao, Q. (2017). Carbon allowance auction d esign of China's emissions trading scheme: A multi-agent-based approach. Energy Policy *102*, 30–40. https://doi.org/10.1016/j.enpol.2016.11.041.
6. Liang, Y., Yu, B., and Wang, L. (2019). Costs and benefits of renewable energy development in China's power industry. Renewable Energy *131*, 70 0–712. https://doi.org/10.1016/j.renene.2018.07.079.
7. Tang, B. (2018). How to peak carbon emissions in China's power sector_ A regional perspective. Energy Policy.
8. Rubin, E.S., Azevedo, I.M.L., Jaramillo, P., and Yeh, S. (2015). A review of learning rates for electricity supply technologies. Energy Policy *86*, 198 –218. https://doi.org/10.1016/j.enpol.2015.06.011.
9. Chen, H. (2018). Electricity System Planning and Decision-making Method ology and Its Applications During the Low-carbon Transition.
10. Wiebe, K.S., and Lutz, C. (2016). Endogenous technological change and th e policy mix in renewable power generation. Renewable and Sustainable E nergy Reviews *60*, 739–751. https://doi.org/10.1016/j.rser.2015.12.176.
11. Rubin, E.S., Azevedo, I.M.L., Jaramillo, P., and Yeh, S. (2015). A review of learning rates for electricity supply technologies. Energy Policy *86*, 198 –218. https://doi.org/10.1016/j.enpol.2015.06.011.
12. Wei, Y., Zhu, R., and Tan, L. (2022). Emission trading scheme, technologi cal innovation, and competitiveness: Evidence from China's thermal power enterprises. Journal of Environmental Management *320*, 115874. https://doi. org/10.1016/j.jenvman.2022.115874.
